# Equine mesenchymal stromal cells and embryo-derived stem cells are immune privileged *in vitro*

**DOI:** 10.1186/scrt479

**Published:** 2014-07-30

**Authors:** Yasmin Z Paterson, Nicola Rash, Elaine R Garvican, Romain Paillot, Deborah J Guest

**Affiliations:** Animal Health Trust, Lanwades Park, Kentford, Newmarket, Suffolk, CB8 7UU UK

## Abstract

**Introduction:**

Autologous mesenchymal stem cells (MSCs) are an attractive concept in regenerative medicine, but their mechanism of action remains poorly defined. No immune response is reported after *in vivo* injection of allogeneic equine MSCs or embryo-derived stem cells (ESCs) into the equine tendon, which may be due to the cells’ immune-privileged properties. This study further investigates these properties to determine their potential for clinical application in other tissues.

**Methods:**

Mitomycin C-treated MSCs, ESCs, or differentiated ESCs (dESCs) were cultured with allogeneic equine peripheral blood mononuclear cells (PBMCs), and their effect on PBMC proliferation, in the presence or absence of interferon-gamma (IFN-γ) was determined. MSCs and super-antigen (sAg)-stimulated PBMCs were co-cultured directly or indirectly in transwells, and PBMC proliferation examined. Media from MSC culture were harvested and used for PBMC culture; subsequent PBMC proliferation and gene expression were evaluated and media assayed for IFN-γ, tumor necrosis factor alpha (TNF-α), and interleukin (IL)-10 and IL-6 proteins with enzyme-linked immunosorbent assay (ELISA).

**Results:**

Co-culture of PBMCs with ESCs or dESCs did not affect baseline proliferation, whereas co-culture with MSCs significantly suppressed baseline proliferation. Stimulation of PBMC proliferation by using super-antigens (sAgs) was also suppressed by co-culture with MSCs. Inhibition was greatest with direct contact, but significant inhibition was produced in transwell culture and by using MSC-conditioned media, suggesting that soluble factors play a role in MSC-mediated immune suppression. The MSCs constitutively secrete IL-6, even in the absence of co-culture with PBMCs. MSC-conditioned media also brought about a change in the cytokine-expression profile of sAg-stimulated PBMCs, significantly reducing PBMC expression of IL-6, IFN-γ, and TNF-α.

**Conclusions:**

Equine MSCs and ESCs possess a degree of innate immune privilege, and MSCs secrete soluble factors that suppress PBMC proliferation and alter cytokine expression. These properties may make possible the future clinical use of allogeneic stem cells to help standardize and broaden the scope of treatment of tissue injuries.

## Introduction

The use of autologous mesenchymal stromal/stem cells (MSCs) in clinical practice to aid tendon regeneration in horses [1] has gained popularity and acceptance in the last decade. Results from the clinical and experimental use of MSCs in regenerative medicine [2–5] have been promising, but details of the cellular mechanism of action remain unknown. Previous work has shown that MSC survival after injection into the injured tendon is low (39% retention 6 hours after intra-arterial regional limb perfusion, 28% retention after intravenous administration [6, 7], and <5% survival 10 days after implantation [8]), which suggests that their beneficial effects are not brought about solely through their direct differentiation into tendon cells.

This theory is supported by the results of other studies using MSCs, which have shown them to function through trophic effects on endogenous cells [9] rather than through directed differentiation. MSCs have also been shown to have immunomodulatory properties both *in vivo*[10] and *in vitro*[5, 11], and these attributes may render them potential candidates for the treatment of inflammatory conditions.

MSCs are currently being used in human trials to reduce inflammation in a range of conditions such as spinal cord injury [12], knee osteoarthritis [13], and liver failure [14]. In horses, immunomodulatory properties may suggest that equine MSCs could have potential use in the future treatment of inflammatory conditions such as osteoarthritis [15] or inflammatory airway disease.

Current clinical practice uses autologous MSCs. However, this approach requires aspiration of bone marrow from every horse, an invasive technique with the risk of potential complications, such as pneumopericardium [16]. After aspiration, culture expansion of MSCs to obtain sufficient numbers for clinical use can take up to 4 weeks, precluding the treatment of acute injuries during the initial inflammatory peak. In addition, treatment of each individual by using MSCs obtained from that individual makes it impossible to standardize treatment fully. In the horse, embryo-derived stem cells (ESCs) [17, 18] are also being investigated for their potential to aid tendon regeneration [8, 19] by providing an allogeneic source of cells that could be standardized to provide an "off-the-shelf" treatment. Equine ESCs have a high survival in the injured horse tendon without inducing any apparent immune response [8]. Furthermore, they appear to undergo some degree of tenocyte differentiation, which has also been demonstrated *in vitro* in response to TGF-β3 and 3D culture [20]. However, whether equine ESCs would be immune privileged after transplantation and differentiation into other tissues remains unknown.

Many of the limitations of current autologous treatment could be overcome by the use of allogeneic MSCs or ESCs. Work in other species has demonstrated that ESCs are immune privileged to some degree, although they may ultimately still be recognized and consequently rejected by the immune system [21–24]. Likewise, both human and equine MSCs possess some ability to modulate an immune response [25–29], although their precise mechanism of action is largely unknown. It was previously shown that allogeneic equine MSCs can be transplanted into the injured tendon (single dose) [8], injected intradermally (two doses, 3 to 4 weeks apart) [30] or intraarticularly (single dose) [31] without eliciting an apparent immune response. Additionally, no changes in cellular or humoral immunity parameters were reported after intravenous injection of allogeneic MSCs into six healthy horses [32]. Recent *in vitro* results showed that equine MSCs do not significantly alter the baseline proliferation of nonactivated T cells [28, 30], but that they can decrease the proliferation of stimulated T cells [28]. When in co-culture with stimulated T cells, the MSCs were found to produce increased amounts of prostaglandin and IL-6 and to decrease the production of TNF-α and IFN-γ by the T cells. Secreted prostaglandin E_2_ recently was shown to be involved in equine MSC-mediated T-cell suppression [29].

To determine whether equine ESCs have the potential to be used in the treatment of injuries to tissue other than tendon, where cell replacement may be beneficial, we determined whether they were immune privileged *in vitro* by performing co-cultures with equine peripheral blood mononuclear cells (PBMCs). We also determined whether equine MSCs had immune-modulatory properties and if this was dependent on direct cell-to-cell contact. In addition, we examined the resulting cytokine-expression profile of PBMCs after culture in MSC-conditioned media.

## Methods

All of the work described was performed with the approval of the Animal Health Trust Ethical Review Committee and, where live experimental animals were involved, under UK Home Office Licence.

### Stem cell culture

Three lines of previously characterized ESCs [17, 18] were used in this study. ESCs were cultured on mitotically inactivated mouse embryonic fibroblasts at 37.5°C, 5% CO_2_, in ESC medium (DMEM/F12 containing 15% fetal bovine serum, 2 m*M* L-glutamine, 1% nonessential amino acids, 1 m*M* sodium pyruvate, 0.1 m*M* 2-mercaptoethanol (all from Invitrogen, Renfrewshire, UK), and 1,000 units/ml leukemia inhibitory factor (LIF) (Sigma, Dorset, UK)). ESCs were passaged mechanically every 5 to 7 days in the presence of Effectine (PAA Laboratories, Somerset, UK). To induce differentiation, ESCs were passaged into conditions without feeder cells in the absence of LIF.

MSCs were derived from bone marrow aspirates taken from live Thoroughbred geldings, as described previously [33] or from Welsh mountain ponies immediately after euthanasia for reasons unrelated to this study. All MSCs were characterized according to their expression of surface antigens and tri-lineage differentiation, as described previously [34]. Two 10.5-ml aliquots of bone marrow were aspirated into heparin (Sigma). Bone marrow was centrifuged through histopaque (Sigma), and the buffy layer of mononuclear cells was collected and washed in culture medium (DMEM supplemented with 10% fetal calf serum, 2 m*M* L-glutamine, 100 U/ml penicillin, and 100 μg/ml streptomycin (all from Invitrogen, Paisley, UK)) before plating all recovered cells in 10 ml medium onto a 10-cm plate for incubation at 37.5°C, 5% CO_2_. Medium was replaced every 2 to 3 days to remove nonadherent cells, and adherent cells were passaged with 0.25% trypsin-EDTA (Sigma) every 3 to 7 days and replated at a seeding density of approximately 10,000 cells/cm^2^. Cells at passages 2 to 4 were frozen in liquid nitrogen until needed for culture.

Equine MSCs and ESCs were plated in standard growth media and allowed to attach overnight before the addition of 100 ng/ml equine IFN-γ (R&D Systems, Abingdon, UK) for 72 h.

### Peripheral blood mononuclear cell isolation, culture, and stimulation with mitogen or *Streptococcus equi*superantigens

Peripheral blood mononuclear cells (PBMCs) were purified by centrifugation on Ficoll-Hypaque (Amersham Biosciences, Uppsala, Sweden), as previously described [35]. Blood was collected from Welsh mountain ponies (not the same ponies from which MSCs were obtained) for reasons unrelated to this study, and only excess PBMCs were used for the following experiments. Pellets of excess PBMCs were resuspended in 1 ml of PBMC media (RPMI-1630 (Sigma) with 10% heat-inactivated fetal calf serum, 100 U/ml penicillin, and 100 μg/ml streptomycin, 2 m*M* L-glutamine, and 55 μ*M* 2β-mercaptoethanol (as before)) and numerated.

For culture-stimulation experiments, PBMCs were treated with phytohemagglutinin (PHA) (Sigma) at a concentration of 5 μg/ml or *Streptococcus equi* superantigens (sAgs) (SeeM, SeeL, SeeI, and SeeH, each at a final concentration of 0.125 μg/ml (AHT Bacteriology Unit)), as previously described [36].

### Mixed lymphocyte reactions and co-cultures

For MLRs, mitomycin C (MMC)-treated MSCs and differentiated ESCs (125 μg/ml MMC for 2-hour incubation) were cultured in 96-well plates in the presence of nonstimulated effector PBMCs at a ratio of 1:2 stem cells/PBMCs. For the negative control, MMC-treated PBMCs (50 μg/ml MMC for 30 minutes) were washed and cultured with homologous effector PBMCs. For the positive control, MMC-treated PBMCs were cultured with heterologous effector PBMCs, both at ratios of 1:2 stimulator/effector PBMCs. After 5 days, PBMCs were treated with radioactive thymidine (^3^H thymidine) (GE Healthcare Bio-sciences) at a final concentration of 0.5 μCi per well and incubated at 37°C, 5% CO_2_ for 16 to 18 hours. Cells were harvested and numerated. Three replicates using three lines of cells were performed.

For the ESCs co-cultures were also performed in six-well plates because undifferentiated ESCs grow in colonies and require monolayer culture on a feeder layer to maintain their undifferentiated state. Therefore MMC-treated undifferentiated ESCs and differentiated ESCs were cultured with nonstimulated effector PBMCs at various ratios ranging from 1:25 to 1:100 in six-well plates. After 5 days, the proliferation of the PBMCs was determined by using ^3^H-thymidine incorporation. Six replicates with three lines of ESCs were performed.

To determine the immune modulatory properties of MSCs, MMC-treated MSCs were co-cultured with allogeneic PHA or sAg-stimulated PBMCs in six-well plates at ratios of 1:10 to 1:400. After 3 days, PBMC numbers were determined by using ^3^H-thymidine incorporation, and a percentage inhibition value for proliferation was calculated by using the formula: 100-((condition-NAx100)/(sAg-NA)) (where NA is nonactivated PBMCs, and sAg is sAg-stimulated PBMCs). Three replicates using MSCs isolated from three different horses were performed.

### Transwell cultures

Six-well plate transwell cultures with a 0.4 μ*M* membrane pore size (Corning, Costar, Cambridge, MA, USA) were used to separate the MSCs physically from the sAg-stimulated PBMCs. PBMCs at a concentration of 10 × 10^6^ cells/ml were stimulated with sAg, as previously described, and co-cultured with 1 × 10^5^ MMC-treated MSCs, with the PBMCs in the inner chamber of the well and the adherent MSCs in the outer chamber. After 3 days, the PBMCs were quantified by using ^3^H-thymidine incorporation. Three replicates with MSCs isolated from three different horses were performed.

### MSC-conditioned media

MSCs were cultured on 10-cm plates until 70% to 80% confluent. Media were removed and replaced with PBMC medium, which was then harvested after 24, 48, and 72 hours. This supernatant, termed "MSC-conditioned medium," was then filtered through a 0.22-μm filter (Nalgene, UK) and maintained at 4°C before use. sAg-stimulated PBMCs and nonactivated PBMCs were incubated for 3 days with conditioned media from each time point. After incubation, the PBMCs were either quantified by using ^3^Hthymidine incorporation or centrifuged to pellet the cells, after which the supernatant was maintained at -20°C until use in ELISAs, and PBMCs were resuspended in 1 ml TRIzol (Ambion, Paisley, UK). Three replicates using MSCs isolated from three different horses were performed.

### Immunohistochemistry

MSCs or ESCs were cultured on gelatin-coated (Sigma, Dorset, UK) coverslips with or without the addition of 100 ng/ml equine IFN-γ (R + D Systems, Abington, UK) for 72 hours, fixed in 3% paraformaldehyde (in PBS) for 20 minutes at room temperature, and permeabilized for 1 hour with 0.1% Triton-X-100 at room temperature. Primary antibody incubations with mouse anti-MHCI 1:200 and mouse anti-MHCII 1:200 (both VMRD, Pullman, WA, USA) were carried out overnight at 4°C before detection with a secondary antibody goat anti-mouse FITC 1:200 (Abcam, Cambridgeshire, UK). Three replicates for each cell type were performed by using cells isolated from different animals.

### Quantitative reverse transcription PCR

PBMC total RNA was extracted by using TRIzol reagent followed by RNA isolation with RNeasy minicolumns and reagents (Qiagen Ltd., Crawley, Surrey, UK). Residual DNA was removed by performing an on-column DNAse digestion by using an RNase-free DNAse kit (Qiagen Ltd.), after which cDNA was synthesized by using the Superscript First-Strand Synthesis System for RT-PCR (Invitrogen). One-microliter aliquots of cDNA diluted 1:1,000 were amplified by polymerase chain reaction on a Quantica qPCR instrument (Techne), by using gene-specific primers (Table 1) in a 25-μl reaction volume with a SYBR Green Core Kit for detection (Eurogentec, Seraing, Belgium). Relative expression levels were normalized with the housekeeping gene 18S and calculated with the 2^-ΔΔCT^ method [37]. Three replicates using conditioned media from MSCs isolated from three different horses were performed.

**Table 1:** **Primers used for RT-PCR** [38]

Primer	Forward (5′-3′)	Reverse (5′-3′)
18S	ATGCGGCGGCGTTATTCC	GCTATCAATCTGTCAACTCCT
TNF-α	AAAGGACATCATGAGCACTGAAAG	GGGCCCCCTGCCTTCT
IFN-γ	CTACCTATTACTGCCAGGCCG	TCCAGGAAAAGAGGCCCAC
IL-6	TGCTGGCTAAGCTGCATTCA	GGAAATCCTCAAGGCTTCGAA
IL-8	TTGGCCGTCTTCCTGCTTT	GGTTTGGAGTGCGTCTTGATG
IP-10	CCTCCAGTTGCAGCACCAT	TTCCTTGAGTTCCACTCAGAGTCA
CCL5	CACTGCCACCTTCTGCACTC	CGGGAGATGTAGGCAAAGCA

### ELISA assay

The IL-6, IL-10, IFN-γ, and TNF-α concentrations in MSC-conditioned media were measured both before and after PBMC culture, by using species-specific competitive inhibition ELISAs (BlueGene, Shanghai, China, and R&D Systems, Abingdon, UK) measured in duplicate on a microplate absorbance reader (ThermoMax Technologies, Columbia, MD, USA). Three replicates with conditioned media from MSCs isolated from three different horses were performed.

### Statistical analysis

Data were analyzed by using one-way ANOVA with *post hoc* Tukey where appropriate (SPSS, IBM). Paired data were compared by using a Student *t* test. A *P* value of <0.05 was considered significant.

## Results

### Equine ESCs do not induce the proliferation of equine PBMCs, even after differentiation and pretreatment with IFN-γ

**Figure 1:**
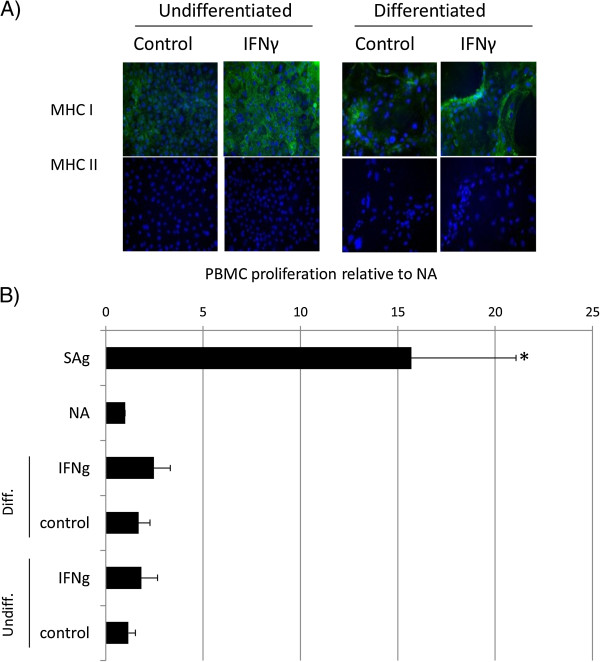
**Proliferation of equine PBMCs is not induced by co-culture with equine ESCs. (A)** Immunocytochemical staining of IFN-γ-treated embryo-derived stem cells (ESCs) for MHC I and MHC II. Cell nuclei are indicated by blue Dapi staining, and expressed MHC I or II proteins, by green staining. Representative images from one of three replicates are shown. **(B)** Relative proliferation of peripheral blood mononuclear cells (PBMCs) to undifferentiated (ESCs) and differentiated (dES) ESCs cultured in the presence and absence of IFN-γ, where NA is baseline, nonactivated PBMC proliferation; sAg is superantigen-stimulated PBMCs (positive control); IFN-γ is 72-hour pretreated undifferentiated or differentiated ESC. *Results significantly different relative proliferation when compared with NA PBMCs (*P* < 0.05). Error bars represent the standard error of seven individual experimental repeats using three different cell lines.

IFN-γ pretreatment of ESCs qualitatively increased the intensity of MHC I staining on both undifferentiated and differentiated cells. However, no induction of MHC II antigens was observed (Figure 1A). The proliferative response of equine PBMCs was measured after co-culture with either undifferentiated ESCs or ESCs that had undergone spontaneous differentiation for 7 days. No increase in PBMC proliferation was observed, even after differentiation (Figure 1B). MHC I upregulation by IFN-γ did not cause ESCs (either undifferentiated or differentiated) to produce a proliferative response from allogeneic PBMCs (Figure 1B).

### Equine bone marrow-derived MSCs suppress background proliferation of unstimulated PBMCs even after pretreatment with IFN-γ

**Figure 2:**
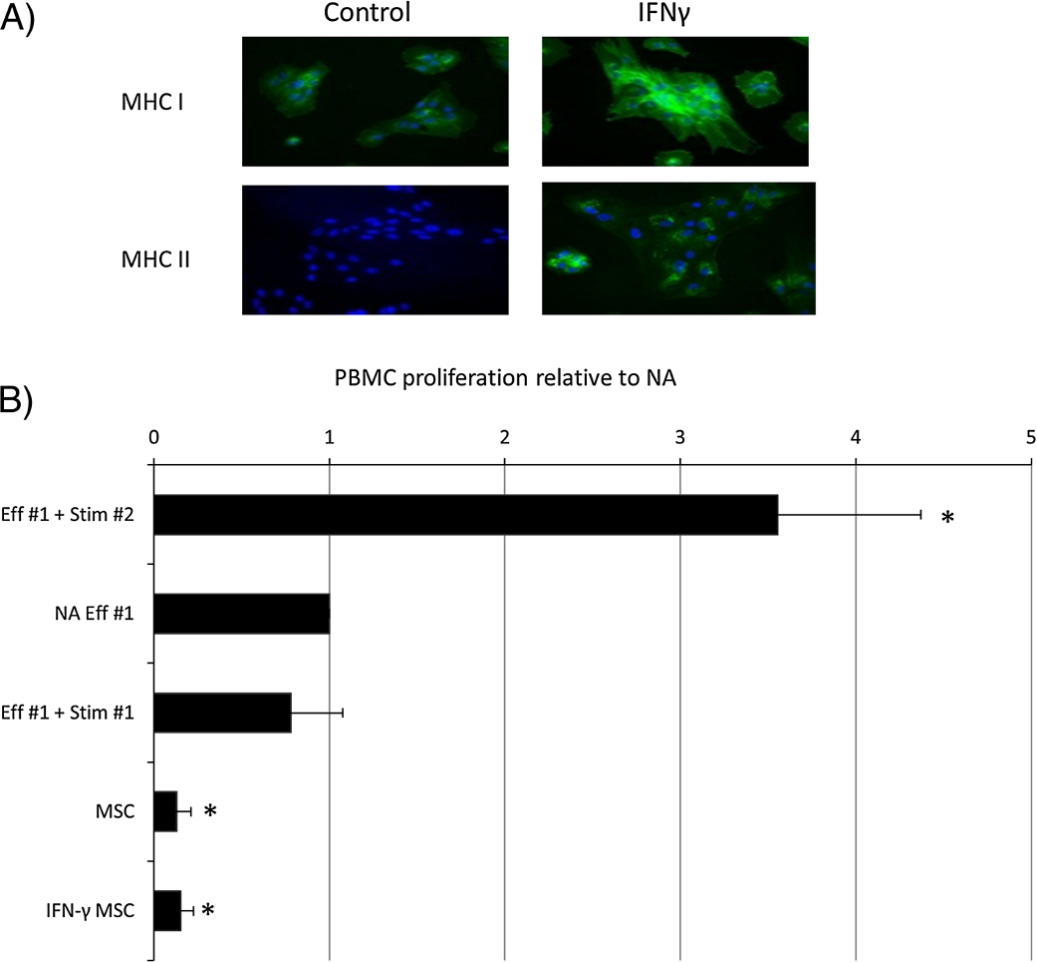
**Proliferation of equine PBMCs is suppressed by co-culture with equine MSCs. (A)** Immunocytochemical staining of IFN-γ-treated mesenchymal stem cells (MSCs) for MHC I and MHC II. Cell nuclei are indicated by blue Dapi staining, and expressed MHC I or II proteins, by green staining. Data show representative images from one of three replicates. **(B)** Relative proliferation of effector (Eff#1) peripheral blood mononuclear cells (PBMCs) to mesenchymal stem cells (MSCs) cultured in the presence and absence of IFN-γ. Autologous and allogeneic stimulator cells are used as negative and positive controls (Stim #1 and Stim #2, respectively). *Results significantly different from those for nonactivated (NA) PBMCs (*P* < 0.05). Error bars represent the standard error of six individual experimental repeats by using three different cell lines.

MSCs express MHC class I antigens, but no MHC class II antigens are detected by using immunocytochemistry. After exposure to IFN-γ, the intensity of MHC I staining is qualitatively increased, and MHC II expression is induced (Figure 2A and [34]). When directly co-cultured with bone marrow-derived MSCs, the baseline level of PBMC proliferation is significantly reduced to approximately 10% of the baseline (Figure 2B; *P* = 0.00001). This finding is in contrast to that observed with ESCs. Furthermore, after treatment with IFN-γ, MSCs continue to decrease significantly the baseline level of PBMC proliferation (Figure 2B; *P* = 0.000002) to a level similar to that of nontreated MSCs with proliferation-inhibition values of 82.5% and 87.7%, respectively.

### Equine bone marrow-derived MSCs suppress PBMCs activated with either PHA or sAgs

**Figure 3:**
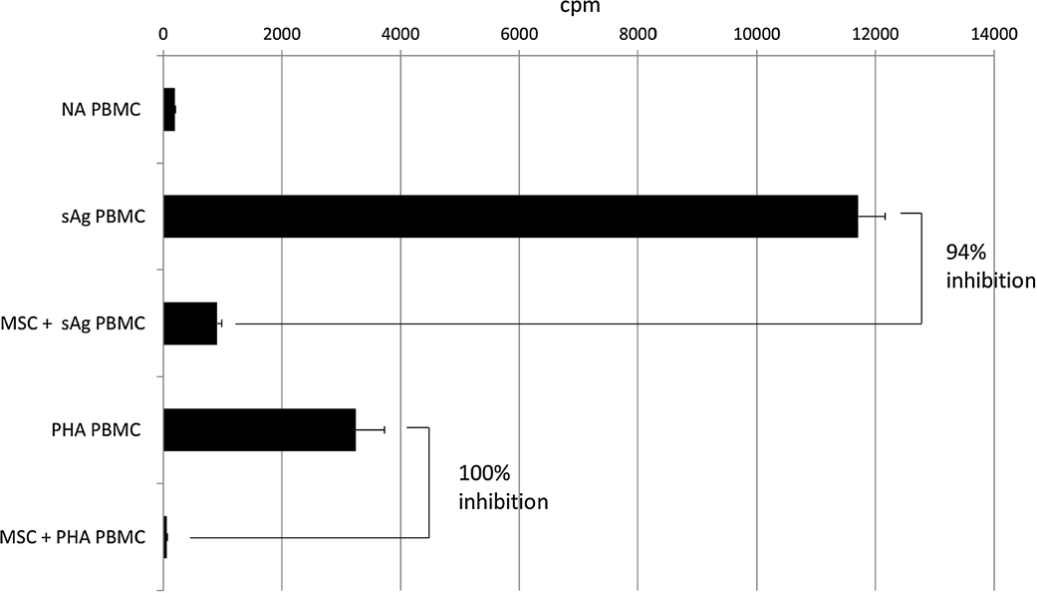
**Proliferation of peripheral blood mononuclear cells (PBMCs) is increased with either** ***S. equi*** **super-antigens (sAg) or phytohemagglutinin (PHA) compared with nonactivated (NA) PBMCs and is inhibited by co-culture with allogeneic mesenchymal stem cells (MSCs).** Graph depicts radioactive thymidine (^3^H-thymidine) counts per minute (cpm) as a measure of cell proliferation. Error bars represent the standard deviation of triplicate wells of the same experiment, which is representative of the three replicates.

Phytohemagglutinin (PHA) is commonly used to stimulate PBMC proliferation [39]. We previously demonstrated that streptococcal sAgs can also lead to PBMC proliferation [36]. By using either mitogen to stimulate PBMC proliferation, we found that co-culture with MSCs results in the inhibition of this proliferation (Figure 3). However, because the use of sAgs results in a greater, more consistent induction of PBMC proliferation, sAgs were used in all subsequent experiments.

### MSCs suppress the proliferation of activated PBMCs to equivalent levels independent of the ratio

**Figure 4:**
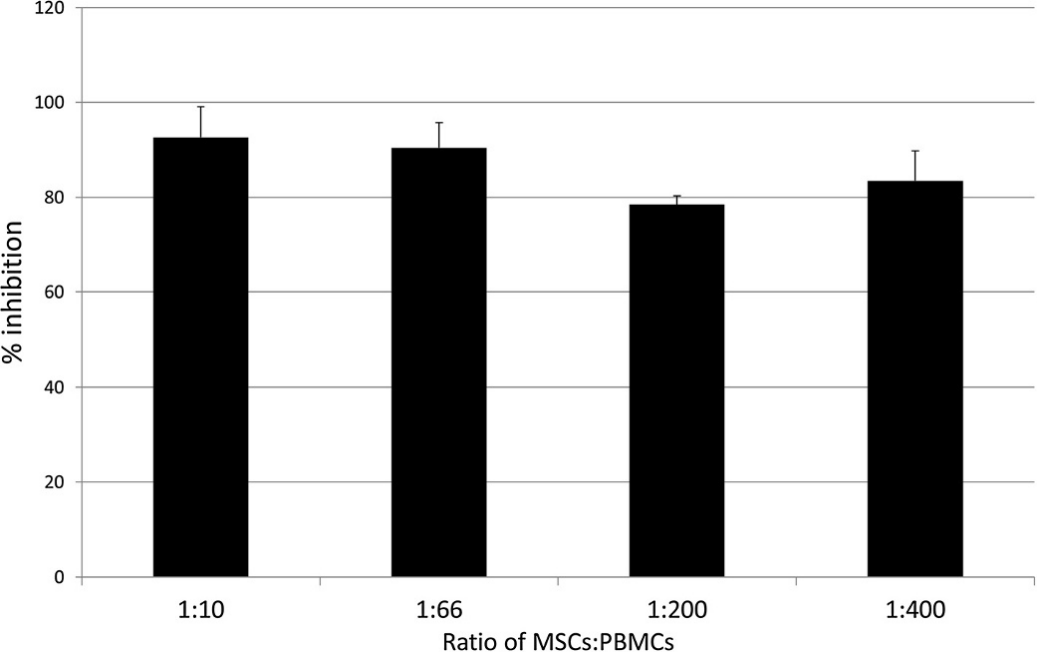
**Percentage inhibition of** ***S. equi*** **superantigen (sAg)-induced peripheral blood mononuclear cells (PBMCs) proliferation when co-cultured with mesenchymal stem cells (MSCs) at ratios of 1:10, 1:66, 1:200, and 1:400 (MSC to PBMC).** Error bars represent the standard error of the mean of three biologic repeats. No significant differences are observed between the ratios (*P* > 0.05).

To determine whether the suppressive effects of the MSCs are lost with an increasing MSC-to-PBMC ratio, MSCs were co-cultured with sAg-activated PBMCs at ratios from 1:10 to 1:400. No significant differences were observed in the percentage inhibition within the tested ratio range, with inhibition remaining consistently high, at 79% to 93% (Figure 4; *P* = 0.28).

### Equine bone marrow-derived MSCs secrete soluble factors that suppress activated PBMCs

**Figure 5:**
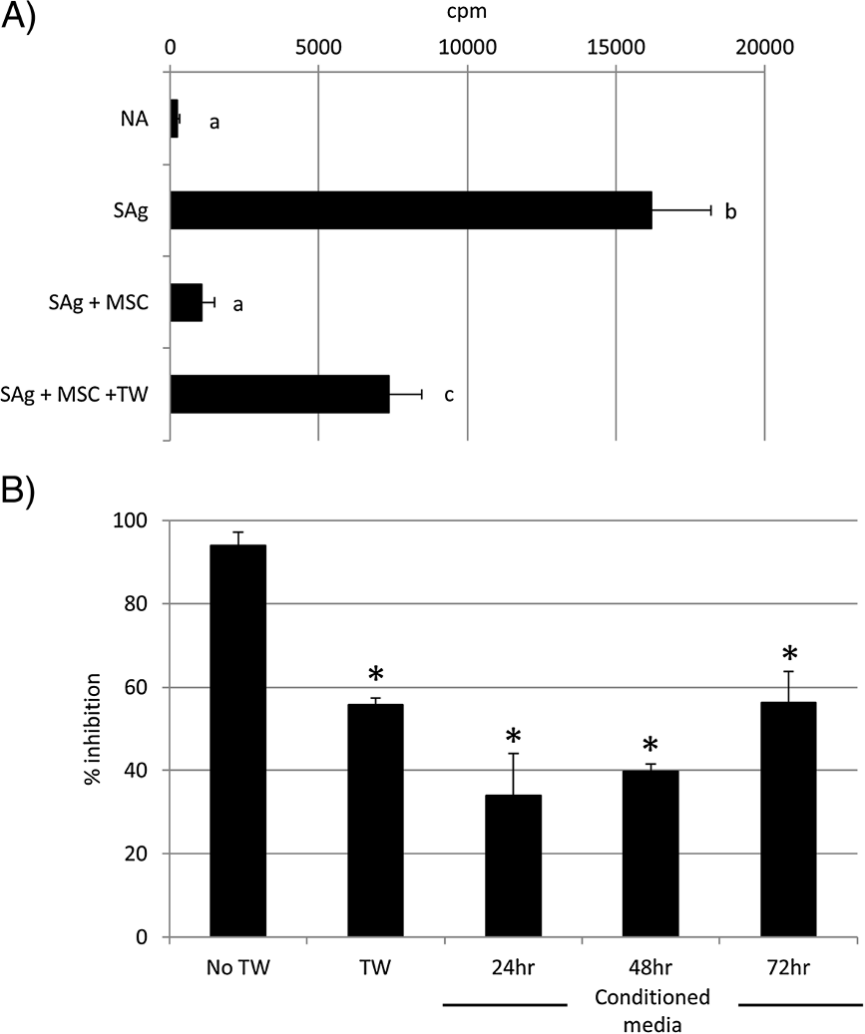
**Soluble factors are involved in MSC-mediated PMBC suppression. (A)** Mesenchymal stem cells (MSCs) suppress *S. equi* superantigen (sAg)-induced peripheral blood mononuclear cells (PBMCs) proliferation when separated by a transwell membrane (TW). Graph depicts radioactive thymidine (^3^H-thymidine) counts per minute (cpm) as a measure of cell proliferation. Differing letter annotations denote a significantly different mean (ANOVA, all *P* < 0.05). Error bars represent the standard error of the mean of three biologic repeats. **(B)** Exposure to 24-, 48-, and 72-hour mesenchymal stem cell (MSC)-conditioned media suppresses *S. equi* superantigen (sAg)-induced peripheral blood mononuclear cell (PBMC) proliferation, but to a lesser extent than via direct cell-to-cell contact. TW, transwell. *Results significantly different from no transwell (No TW) values (ANOVA; *P* < 0.05). Error bars represent the standard error of the mean of three biologic repeats.

In the absence of direct contact between the MSCs and PBMCs, proliferation of the activated PBMCs was inhibited to a significantly lesser degree than that observed when cells were in direct contact (94% inhibition with direct contact; 55% inhibition in transwell system; *P* = 0.006; Figure 5A).

Furthermore, conditioned media taken from cultures of actively proliferating MSCs cultured under standard conditions was used in the PBMC proliferation assays. Media that had been conditioned for 72 hours was found to bring about a 56% inhibition of proliferation, which was not significantly different from that seen in the presence of a transwell (Figure 5B). This demonstrates that the MSCs release soluble factors, even in the absence of any signals received from the PBMCs.

### The factors released by equine MSCs also cause changes in the cytokine-expression profile and protein production of activated PBMCs

**Figure 6:**
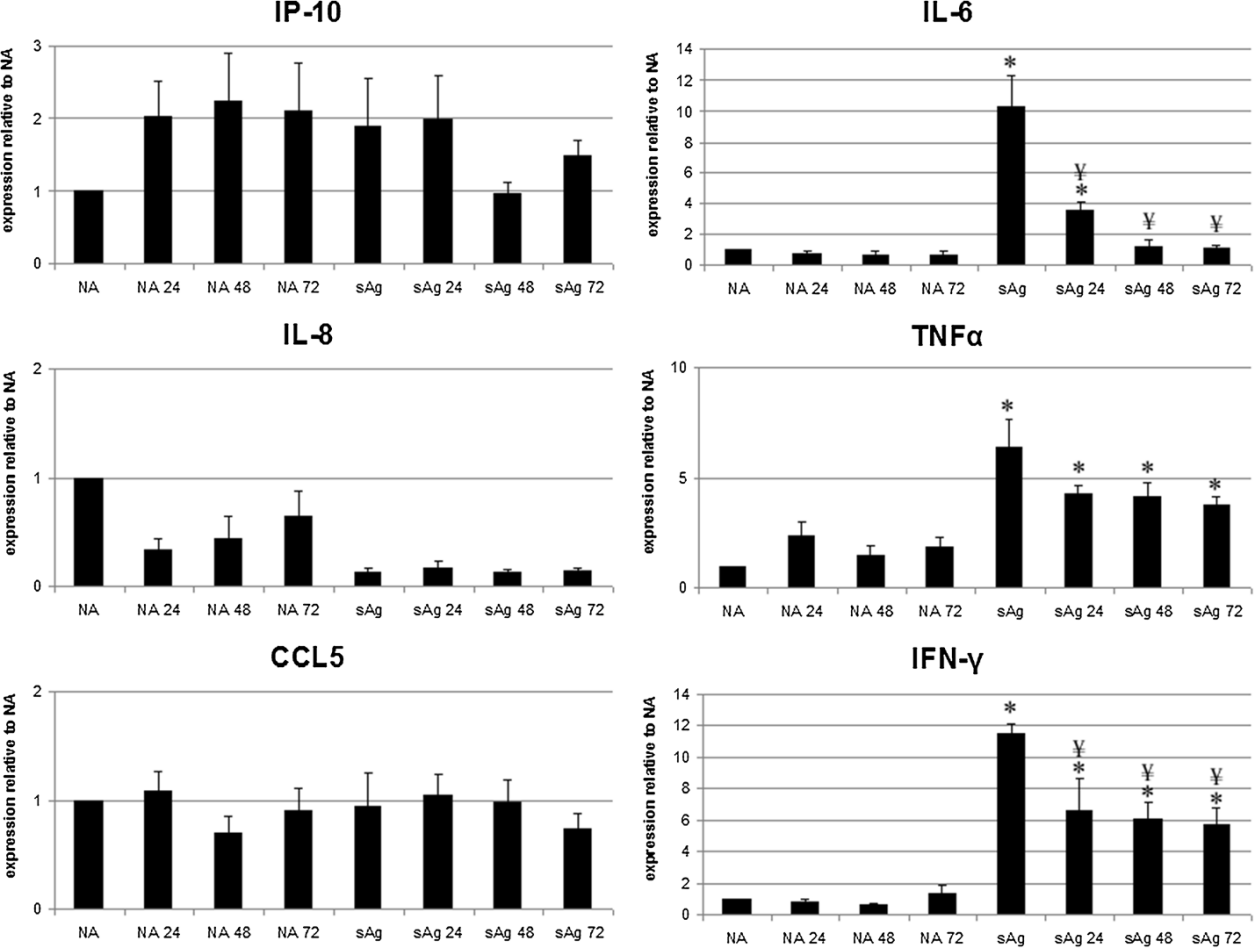
**mRNA expression by nonactivated (NA) and** ***S. equi*** **superantigens (sAg)-stimulated peripheral blood mononuclear cells (PBMCs) after exposure to 24-, 48-, and 72-hour MSC conditioned media (that is, NA24 represents nonactivated PBMCs exposed to 24-hour MSC-conditioned media).** Results represented as change in fold expression relative to NA PBMCs (control conditions), adjusted to the housekeeping gene 18S. Error bars represent the standard error of the mean from three individual experiments using three cell lines. *Results significantly different (*P* < 0.05) from those for NA PBMCs, ¥ denotes values significantly different (*P* < 0.05) from sAg-stimulated PBMCs (sAg, stimulated control).

sAg-stimulation of PBMCs resulted in an increase in expression of IL-6, TNF-α, and IFN-γ mRNA (Figure 6). The presence of 24– to 72-hour MSC-conditioned media significantly reduced this upregulation in IL-6, and IFN-γ mRNA (*P* < 0.01 for each time point). Addition of 48- and 72-hour MSC-conditioned media reduced expression of IL-6 mRNA to a level not significantly different from that of unstimulated PBMCs. The observed reduction in expression of TNF-α mRNA did not reach significance for 24-, 48-, or 72-hour conditioned media (*P* = 0.09, 0.08, 0.07, respectively). No significant changes in gene expression were observed for the chemokines IP-10, IL-8, and CCL5 after activation of PBMCs by sAgs, and incubation with MSC-conditioned media had no additional effect (Figure 6).

**Figure 7:**
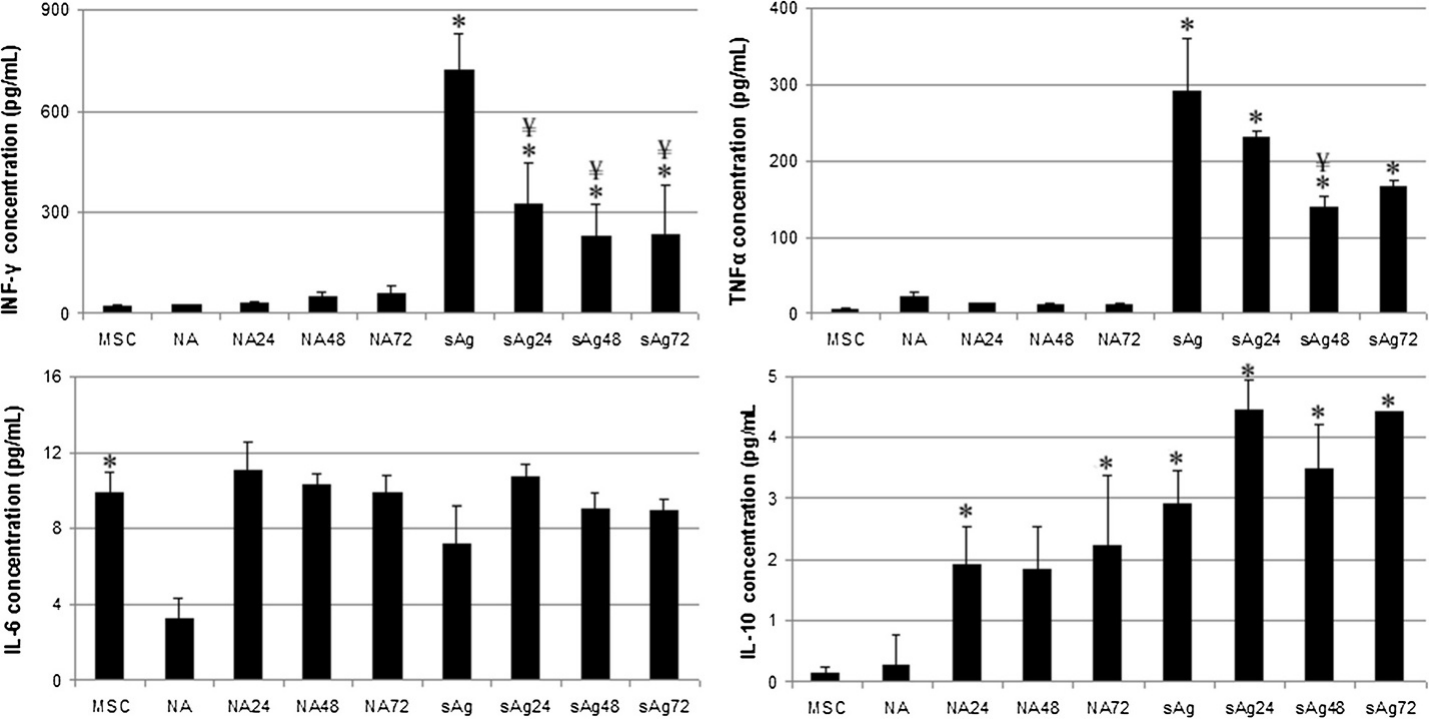
**Concentration of cytokines produced by nonactivated (NA) and** ***S. equi*** **superantigen (sAg)-stimulated peripheral blood mononuclear cells (PBMCs) after exposure to 24-, 48-, and 72-hour MSC-conditioned media, measured with ELISA (that is, NA24 represents nonactivated PBMCs exposed to 24-hour MSC-conditioned media).** Error bars represent the standard error of the mean from three individual experiments using three cell lines. *Results significantly different from those for nonactivated PBMCs (NA), ¥, Values significantly different (*P* < 0.05) from sAg-stimulated PBMCs (sAg).

MSC-conditioned media contained very low concentrations of the proteins IFN-γ, TNF-α, and IL-10 (Figure 7). Media from nonstimulated PBMCs also contained very low concentrations. Production of IL-10 by PBMCs was significantly increased in response to both MSC-conditioned media and sAg stimulation, when compared with nonstimulated PBMCs (*P* < 0.05). Concentrations of IFN-γ and TNF-α in the media of sAg-stimulated PBMCs were significantly higher than those in nonstimulated PBMCs (*P* < 0.05). Culture in MSC-conditioned media significantly reduced this increase for IFN-γ, although concentrations remained significantly higher than those found in nonstimulated controls (*P* < 0.05). Culture in MSC-conditioned media reduced TNF-α concentrations, although this reduction was significant for only 48-hour conditioned media (*P* = 0.04) and not for 72-hour conditioned media (*P* = 0.06) (Figure 7).

In contrast, MSC-conditioned media contained moderate concentrations of IL-6 protein, significantly more than nonstimulated PBMCs, in which expression was very low (*P* < 0.05). Stimulation of PBMCs with sAgs increased IL-6 production, but this failed to reach significance (*P* = 0.08). Culture of PBMCs in MSC-conditioned media resulted in concentrations of IL-6 that were not significantly different from those measured in MSC-conditioned media (Figure 7; *P* < 0.05).

## Discussion

When research into regenerative medicine was in its infancy, it was assumed that the observed clinical benefit of exogenous MSCs was due to *in situ* differentiation of the multipotent cells in a tissue-specific manner. This simplistic view is gradually being replaced with a more sophisticated understanding of the paracrine effect of such cells and their role secreting bioactive molecules and delivering soluble factors to the host cells. This study sheds further light on the immunosuppressive properties of MSCs and ESCs and contributes supporting evidence for the future clinical application of allogeneic cells as a regenerative therapy.

The International Society for Cellular Therapy (ISCT) defined human MSCs as being MHC I positive and MHC II negative [40], and previous reports confirmed that equine MSCs express MHC I [28, 31, 34, 41, 42]. MHC I expression has been reported in 62% to 99% of equine MSCs in these reports, with variability probably due to differences in cell source, passage, antibodies, and laboratory techniques. Multiple studies have shown that equine MSCs do not express MHC II [28, 31, 34, 41]; however, a more recent article suggests that equine MSCs may express variable levels of MHC II, depending on the passage, horse, cell isolation repeat, or culture conditions [42]. Here we demonstrate that both equine ESCs and MSCs, under our culture conditions, express MHC I but not MHC II.

In other species, it is well established that allogeneic MHC-mismatched MSCs do not elicit a proliferative response when cultured with allogeneic PBMCs *in vitro*, resulting instead in a significant reduction in proliferative action [43]. Similar results have recently been demonstrated in the horse [28] in a T-cell-selective population. However, contrary to our findings, background suppression of PBMC proliferation by MSCs was not reported there; we observed significant baseline suppression of PBMC proliferation in culture with MSCs, but not with ESCs. The observed 80% reduction in baseline proliferation is comparable to that observed in canine studies [44].

Our study further supports this concept of immune modulation, with MSCs implementing a reduction in alloreactive lymphocyte proliferation independent of cell ratio (from 1:10 to 1:400 MSC/PBMC). Again, this is in contrast to a previous report in the horse [28] and may reflect differences in the immune cell population (T-cell-selective versus nonenriched PBMC population) or differences in the actual numbers of MSCs used, which has been shown to affect their suppressive effects [45]. We further demonstrated, for the first time in the horse, that MSC-mediated suppression of PBMC proliferation also occurs when using indirect culture or MSC-conditioned media. This supports recent data that demonstrate the involvement of secreted prostaglandin E_2_ in MSC-mediated T-cell suppression [29].

Human adipose-derived multipotent stem cells (AdMSs) demonstrated similar immunomodulatory effects to BMSCs, with both sources suppressing proliferation of stimulated PBMCs and inhibiting monocyte-derived immature dendritic cell differentiation. However, the AdMS cells appeared more potent and secreted greater concentrations of IL-6 and TGF-β1, suggesting that the increased cytokine secretion may contribute to a greater immunomodulatory potency [46]. The proposal that cytokine secretion forms at least part of the mechanism of action is supported in our study by the demonstration that direct contact between cell types is not necessary for the observed inhibitory effect; indeed, simultaneous presence of both MSCs and PBMCs was shown to be not essential, as MSCs are able to suppress alloreactive lymphocytes in both indirect (transwell) and time-lapsed (preconditioning) culture.

Both mRNA and protein expression for the proinflammatory cytokines TNF-α and IFN-γ by activated PBMCs was reduced by culture in MSC-conditioned media. This finding supports previously reported decreases in TNF-α and IFN-γ mRNA expression in human, murine, and equine studies [27, 28, 47, 48], and supports the hypothesis that MSCs promote tissue healing via their ability to decrease immune cell inflammatory signals. Although expression of IL-6 mRNA was similarly decreased in the presence of MSC-conditioned media, the higher baseline production of IL-6 by MSCs may have masked any corresponding downregulation in protein expression by PBMCs. Our finding that equine MSCs constitutively express IL-6 is in contrast to previous reports [28], although in agreement with findings from other species [49]. It has been suggested that IL-6 production by MSCs is a key component of their immune privilege and that postimplantation rejection of allogeneic MSCs may be related to a reduction in cellular IL-6 concentrations [49], although inhibition of IL-6 did not affect equine MSC-mediated T-cell suppression in a recent study [29].

Our study found no increase in chemokine mRNA expression (IP-10, CCL5, IL-8) after sAg activation of PBMCs. In contrast, studies on human allogeneic MSCs and PBMCs demonstrated upregulation of chemokine expression and protein release after stimulation with inflammatory mediators [50]. It is also hypothesized that MSCs promote antiinflammatory signaling, by upregulating key antiinflammatory cytokines such as IL-10 [27, 28, 30], as observed here.

We, and others previously demonstrated that MSCs upregulate MHCI expression and induce expression of MHCII in response to IFN-γ [34, 42]. Although our study did not quantify the level of expression, this would be interesting to explore in future experiments, as our results suggest that although the majority of equine MSCs and ESCs express MHC I under normal conditions, the level of expression is increased after IFN-γ treatment. In addition, IFN-γ acts as a major regulator of the expression of chemokines and their receptors in human MSCs [50]. Although in human tendons, IFN-γ levels remain below detection levels after injury [51], in the injured equine superficial digital flexor tendon, a significant percentage of tenocytes have been shown to be IFN-γ immunopositive [52]. These data confirm that addition of IFN-γ does not reduce the immune privilege of either ESCs or MSCs, although the resultant effect on MHC expression differed (significant upregulation of MHC I, with no effect on MHC II in ESC lines, in comparison with upregulation of both MHC I and II in MSCs).

It has been suggested that low MHC expression results in a reduction of stimulatory signals to aid in evasion of an immune attack [22, 24], forming an additional or alternate mechanism by which MSCs and ESCs were able to avoid stimulation of a proliferative response in PBMCs. Similar findings are attributed to studies on human MSCs [45, 53], but MHC II expression has been associated with MHC-mismatched T-cell proliferation in horses [42]. The haplotypes of the horses used in the current study are unknown, but these conflicting results warrant further research. An absence of co-stimulatory molecules in human MSCs may also help them to evade the immune system [54], and although some of these molecules are expressed at the mRNA level in equine MSCs [41], a lack of equine-specific antibodies has precluded detailed investigations to date.

Equine ESCs have been observed to undergo a degree of tenocyte differentiation after implantation into injured tendon *in vivo* and furthermore, not to elicit an immune response [19]. However, the relative importance of the host tendon tissue in this immune privilege is not known, as equine ESCs have not, to date, been implanted into other tissues. In this study, we demonstrated that equine ESCs do not induce the proliferation of allogeneic PBMCs, even after their spontaneous differentiation. This may in part be due to a lack of MHC II expression by both undifferentiated and differentiated ESCs, even after exposure to IFN-γ and supports results reported in ESCs from other species [24]. When equine ESCs are allowed to undergo spontaneous differentiation, a mixed population of differentiated cell types is produced, which contains cells derived from all three germ layers [17, 18]. The observed lack of induction of PBMC proliferation after *in vitro* differentiation therefore supports further *in vivo* research into their application in other tissues. Unlike, MSCs, equine ESCs did not suppress baseline PBMC proliferation, which may suggest that they do not have the same immunosuppressive properties as MSCs. However, the effect of ESCs on the proliferation of activated PBMCs was not performed in this study and should be addressed in the future.

Furthermore, the effect of repeated doses of stem cells for therapeutic aims is not known and should form part of future work. In baboons, multiple administrations of high doses of allogeneic MSCs reportedly affected alloreactive immune responses without compromising the overall immune system of recipient baboons [55]. The authors concluded that induction of host T-cell hyporesponsiveness to donor alloantigens may facilitate MSC survival. Additionally, equine umbilical cord blood stem cells neither stimulated, nor suppressed, baseline proliferation rates of PBMCs *in vitro*, or after repeated administration *in vivo*[30].

## Conclusion

In conclusion, both MSCs and ESCs are attractive targets for the development of allogeneic cellular therapy. Equine ESCs did not affect baseline PBMC proliferation, even after differentiation and/or MHC upregulation by IFN-γ. In contrast, equine MSCs have a profound suppressive effect on allogeneic lymphocytes, a feature not dependent on MHC expression, suggesting efficacy regardless of donor MHC haplotype. Preservation of this suppressive effect on mitogen-stimulated PBMCs, along with the suppression of pro-inflammatory cytokine production, suggests that clinical delivery into an inflamed environment may also be valuable. MSCs have potential as immune-regulatory tools for the treatment of immune-mediated and inflammatory diseases, such as osteoarthritis and inflammatory airway disease, through their ability to produce immunomodulatory trophic factors.

## Abbreviations

AdMS: Adipose-derived multipotent stem cell
CCL5: chemokine ligand 5
cpm: counts per minute
ELISA: enzyme linked immunosorbent assay
ESC: embryonic stem cell
IFN-γ: interferon gamma
IL-10: interleukin 10
IP-10: interferon gamma-induced protein 10
MHC: major histocompatibility complex
MMC: mitomycin C
MSC: mesenchymal stem cell
NA: nonactivated
PBMC: peripheral blood mononuclear cell
PCR: polymerase chain reaction
sAg: *S. equi* superantigen
TGF-β: transforming growth factor beta
TNF-α: tumor necrosis factor alpha.
